# Supplementary material for: Toward High-Energy-Density Aqueous Lithium-Ion Batteries Using Silver Nanowires as Current Collectors
Source: Molecules. 2022 Nov 25;27(23):8207. doi: 10.3390/molecules27238207 (PMC9736977; doi:10.3390/molecules27238207)
Supplement: Supplementary file 1 [file molecules-27-08207-s001.zip › molecules-2040777-supplementary.pdf]

Supplemental Materials

Toward High-Energy-Density Aqueous Lithium-ion Batteries Using Silver Nanowires as Current Collectors

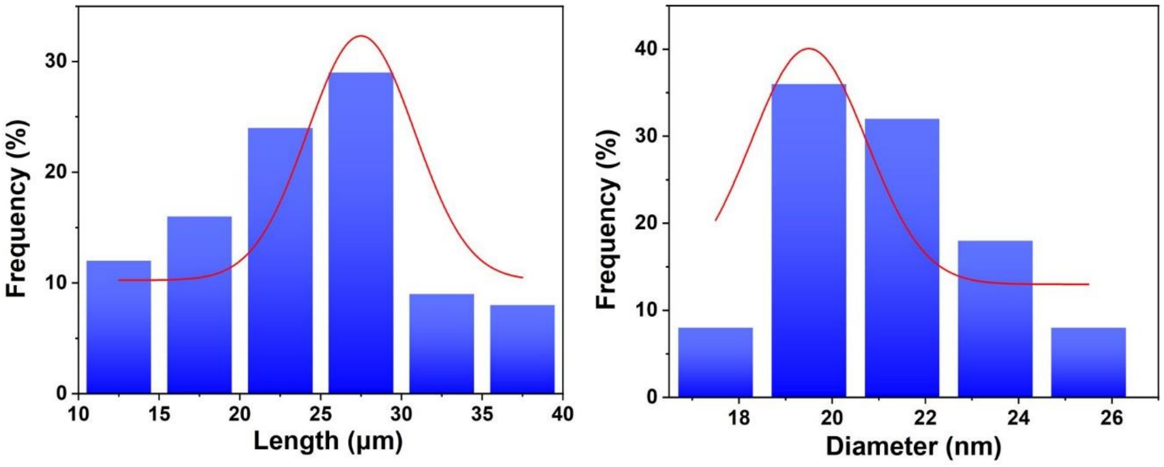

Figure S1. Length and diameter statistics of the AgNWs used in this study.

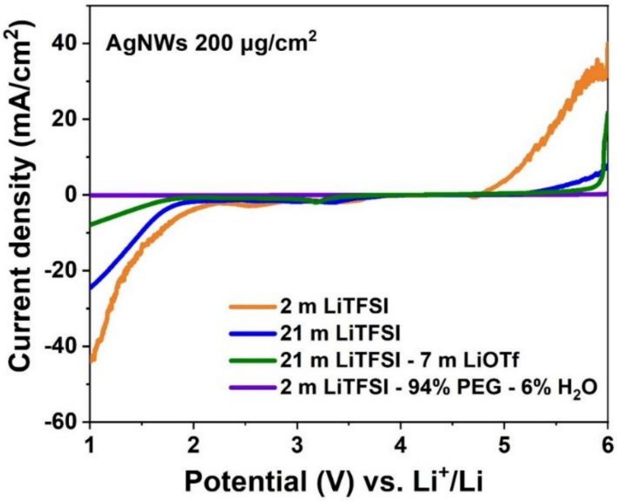

Figure S2. Linear sweep voltammetry (LSV) curves of the AgNW film with areal density of 200 μg/cm^2 under 10 mA/s in various aqueous electrolytes.

Table S1. Electrochemical stability window (ESW)[a] of various current collectors for aqueous lithium-ion batteries.

Current collector	Electrolyte	HER potential (V vs Li^+/Li)	OER potential (V vs Li^+/Li)	ESW (V)	Reference
Au electrode	21 m LiTFSI	2.7	5.2	2.5	**[1]**
Au electrode	2 m LiTFSI–94%PEG–6%H_2O	1.8[b]	5.1[b]	3.3	This work
Al foil	21 m LiTFSI	1.0	6.0	4.7	**[1]**
Al foil	1.2 m LiTFSI	1.8	-	-	**[2]**
Carbon-coated Al foil	2 m LiTFSI–94%PEG–6%H_2O	1.3	4.5	3.2	**[3]**
Al foil	2 m LiTFSI–94%PEG–6%H_2O	1.0[b]	6.0[b]	5.0	This work
AgNWs (100 μg/cm^2)	2 m LiTFSI–94%PEG–6%H_2O	1.0[b]	6.0[b]	5.0	This work
AgNWs (200 μg/cm^2)	2 m LiTFSI–94%PEG–6%H_2O	1.8[b]	5.6[b]	3.8	This work

Stainless steel	21 m LiTFSI	1.9	4.9	3.0	**[4]**
Stainless steel	21 m LiTFSI + 7 LiOTf	1.83	4.9	3.1	**[5]**
Pt	1.2 m LiTFSI	2.35	4.45	2.1	**[2]**

[a] ESW were obtained by calculating the potential difference of OER and HER of the current collectors, which were measured by using CV or LSV tests. [b] Threshold current was selected to be 0.1 mA/cm^2.

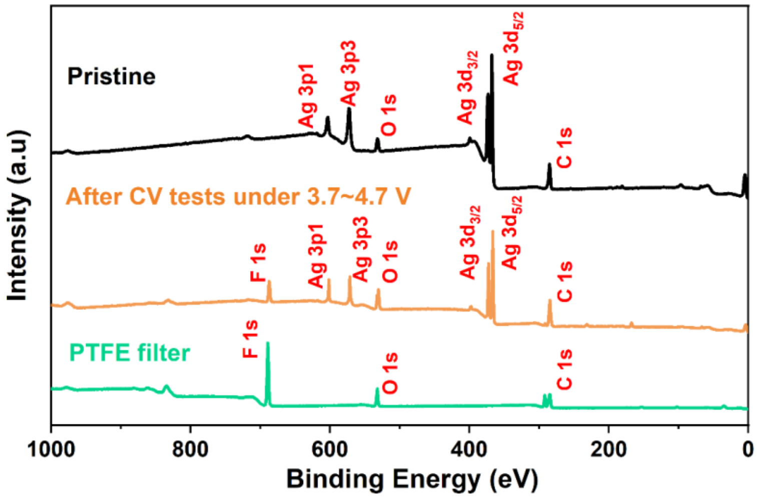

Figure S3. Typical XPS profiles of the AgNW films filtrated on PTFE membranes before and after cyclic voltammetry tests within 3.7-4.7 V vs Li$^+$/Li. The XPS profile of the PTFE filter is also given as a benchmark.

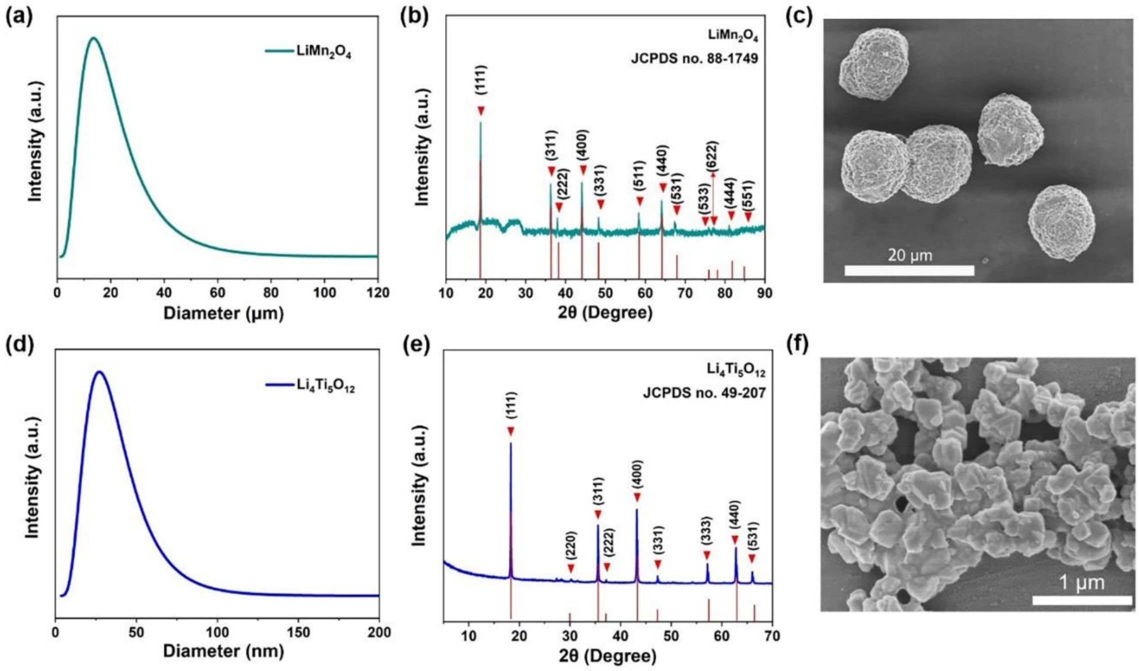

Figure S4. Size distribution, XRD pattern, and typical SEM morphology of the LMO and LTO particles. Top row and bottom row corresponding to the LMO and LTO, respectively.

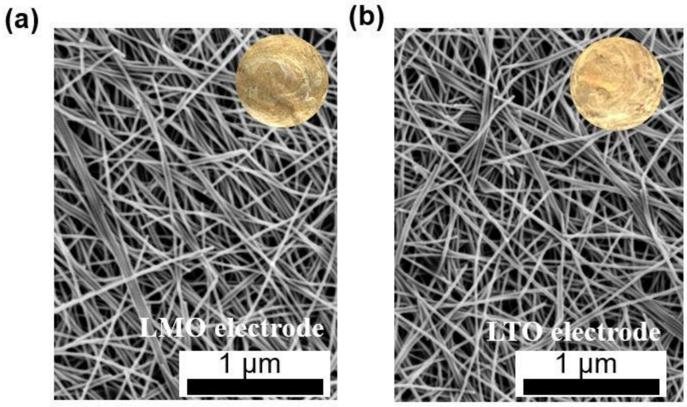

Figure S5. Top-view SEM images of (a) LMO and (b) LTO electrodes at the AgNW-coating side. The insets showing the photos of the electrodes.

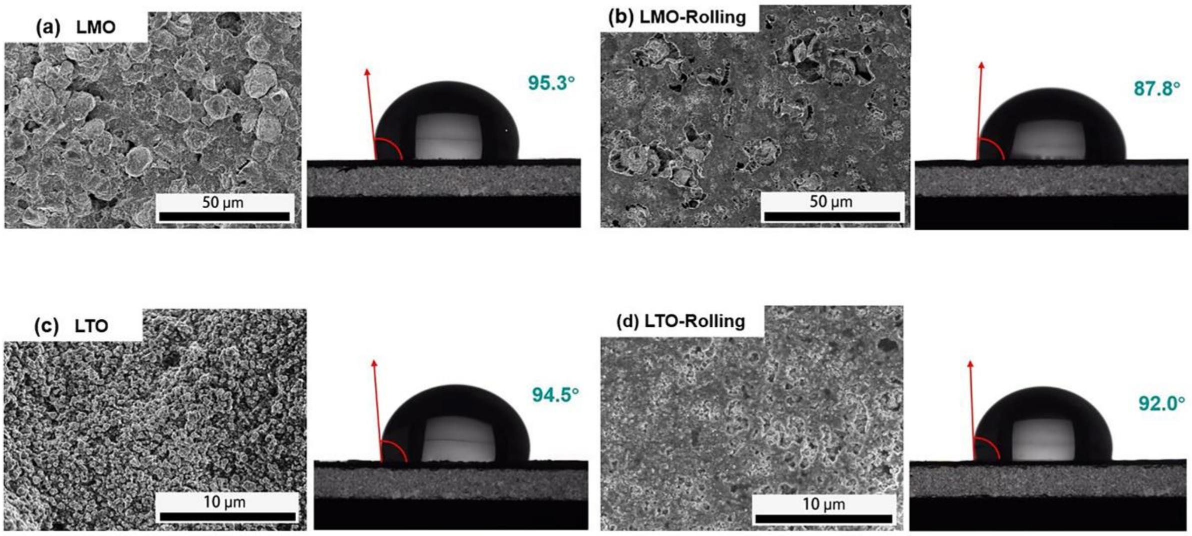

Figure S6. Typical SEM images and static contact angles with AgNW aqueous dispersion of the electrode surfaces before and after press rolling. Top row: the LMO electrodes; bottom row: the LTO electrodes.

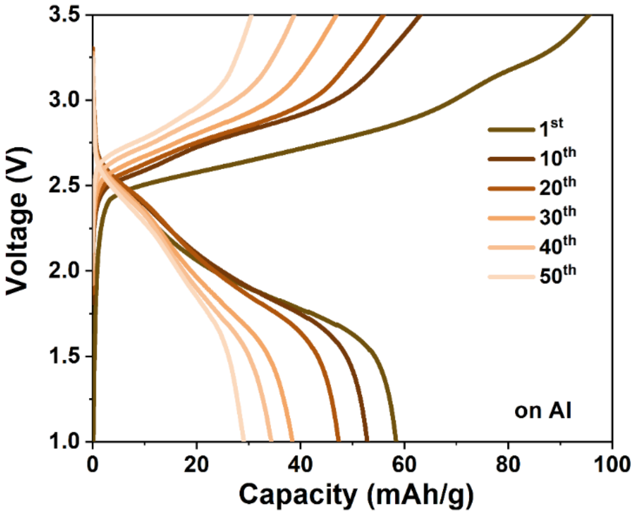

Figure S7. Galvanostatic charge-discharge curves of an aqueous LMO//LTO full cell using Al foil as current collectors cycled at 17.5 mA/g (based on the mass of the LTO).

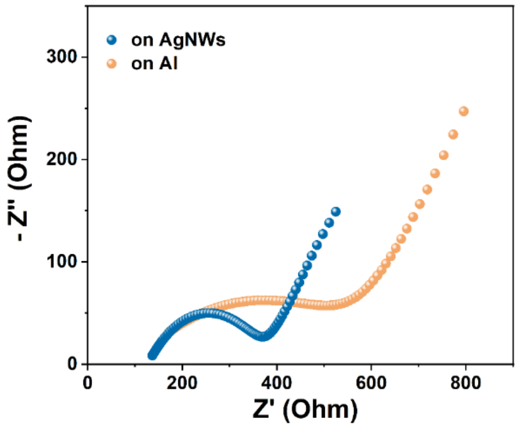

Figure S8. Nyquist plots of the LMO//LTO full cells after 50 charge/discharge cycles under the same current density of 17.5 mA/g (based on the mass of the LTO).

Table S2. Specific capacity and energy density of full cells based on total mass of the electrodes.

**Cathode/Total mass, mg/cm²**	**CC, mg/cm²**	**Anode/Total mass, mg/cm²**	**CC, mg/cm²**	**Total mass of electrodes, mg/cm²**	**Capacity/ mAh/g**	**Voltage/V**	**Energy density/ Wh/kg**	
LMO/KB/PVDF 8.8	AgNWs 0.2	LTO/KB/PVDF 8.5	AgNWs 0.2	17.8	27.7	2.5	69.3	This work
LMO/AB/PVDF 3.8[a]	AB-Al foil 5.1[a]	L-LTO/KB/PVDF 3.1[a]	AB-Al foil 5.1[a]	17.1[a]	16.1	2.5	40.3[a]	**[3]**
LMO/KB/EPR 88.5[a]	Sus grid 25.5[a]	VO_2(B)/KB/EPR 88.5[a]	Sus grid 25.5[a]	228[a]	37.6[a]	1.5	56.4[a]	**[6]**
LFP@C/AB/PTFE 12.5[a]	Sus grid 25.5[a]	LTP/AB/PTFE 12.5[a]	Sus grid 25.5[a]	76[a]	14.6[a]	0.9	13.1[a]	**[7]**
LMO/AB/PTFE 13.3[a]	Ni grid 27.5[a]	MoO_3[c]/AB/PTFE 12.5[a]	Ni grid 27.5[a]	80.8[a]	9.1[a]	1.22	11.1[a]	**[8]**
LMO/KB/PTFE 18.8[a]	Sus grid	Mo_6S_8/KB/PTFE 12.5[a]	Sus grid	-	42	2.0	84	**[4]**
LMO[b]/AB/PTFE 12.5[a]	Sus grid 25.5[a]	LTP/AB/PTFE 15[a]	Sus grid 25.5[a]	78.5[a]	11.0[a]	1.6	17.6[a]	**[9]**
LFP/KB/PTFE	Sus grid	Mo_6S_8/KB/PTFE	Sus grid	-	40.7	1.15	47	**[10]**

[a] Estimated value; [b] $Li_{1.1}Mn_2O_4$; [c] Alfa-MoO_3@PPy; CC: Current collector; AB: Acetylene black; KB: Ketjen black; LMO: $LiMn_2O_4$; LTO: $Li_4Ti_5O_{12}$; L-LTO: $Li_{1.3}Al_{0.3}Ti_{1.7}(PO_4)_3$; LTP: $LiTi_2(PO_4)_3$; LFP: $LiFePO_4$; EPR: Ethylene propylene diene monomer binder.

References

1. Kuhnel, R.S.; Reber, D.; Remhof, A.; Figi, R.; Bleiner, D.; Battaglia, C. "Water-in-salt" electrolytes enable the use of cost-effective aluminum current collectors for aqueous high-voltage batteries. *Chem Commun.* **2016**, *52*, 10435–10438.
2. Yamada, Y.; Usui, K.; Sodeyama, K.; Ko, S.; Tateyama, Y.; Yamada, A. Hydrate-melt electrolytes for high-energy-density aqueous batteries. *Nature Energy.* **2016**, *1*, 16129.
3. Xie, J.; Liang, Z.; Lu, Y.C. Molecular crowding electrolytes for high-voltage aqueous batteries. *Nat Mater.* **2020**, *19*, 1006–1011.
4. Suo, L.M.; Borodin, O.; Gao, T.; Olguin, M.; Ho, J.; Fan, X. L.; Luo, C.; Wang, C.S.; Xu, K. "Water-in-salt" electrolyte enables high-voltage aqueous lithium-ion chemistries. *Science.* **2015**, *350*, 938–943.
5. Suo, L.; Borodin, O.; Sun, W.; Fan, X.; Yang, C.; Wang, F.; Gao, T.; Ma, Z.; Schroeder, M.; von Cresce, A.; et al. Advanced High-Voltage Aqueous Lithium-Ion Battery Enabled by "Water-in-Bisalt" Electrolyte. *Angew. Chem. In.t Ed. Engl.* **2016**, *55*, 7136–7141.
6. Li, W.; Dahn, J.R.; Wainwright, D.S. Rechargeable lithium batteries with aqueous electrolytes. *Science.* **1994**, *264*, 1115–1118.

7. Luo, J.Y.; Cui, W.J.; He, P.; Xia, Y.Y. Raising the cycling stability of aqueous lithium-ion batteries by eliminating oxygen in the electrolyte. *Nat. Chem*. **2010**, *2*, 760–765.
8. Tang, W.; Liu, L.; Zhu, Y.; Sun, H.; Wu, Y.; Zhu, K. An aqueous rechargeable lithium battery of excellent rate capability based on a nanocomposite of MoO3 coated with PPy and LiMn2O4. *Energy Environ. Sci.* **2012**, *5*, 6909–6913.
9. Dong, X.; Chen, L.; Su, X.; Wang, Y.; Xia, Y. Flexible Aqueous Lithium-Ion Battery with High Safety and Large Volumetric Energy Density. *Angew. Chem. Int. Ed. Engl.* **2016**, *55*, 7474–7477.
10. Suo, L.; Han, F.; Fan, X.; Liu, H.; Xu, K.; Wang, C. "Water-in-Salt" electrolytes enable green and safe Li-ion batteries for large scale electric energy storage applications. *J. Mater. Chem. A.* **2016**, *4*, 6639–6644.
